# Supplementary material for: The underappreciated potential of peatlands in global climate change mitigation strategies
Source: Nat Commun. 2018 Mar 14;9:1071. doi: 10.1038/s41467-018-03406-6 (PMC5851997; doi:10.1038/s41467-018-03406-6)
Supplement: Supplementary file 1 — Supplementary Information [file 41467_2018_3406_MOESM1_ESM.pdf]

Supplementary Information to ‘The underappreciated potential of peatlands in global climate change mitigation strategies’

Leifeld, J. [1], Menichetti, L.[1,2]*

*Correspondence to: jens.leifeld@agroscope.admin.ch

1 Agroscope, Climate / Air Pollution Group, Reckenholzstrasse 191, 8046 Zurich, Switzerland

2 now Ecology, SLU (Sveriges Lantbruksuniversitet), Ulls Väg 16, 75651 Uppsala, Sweden

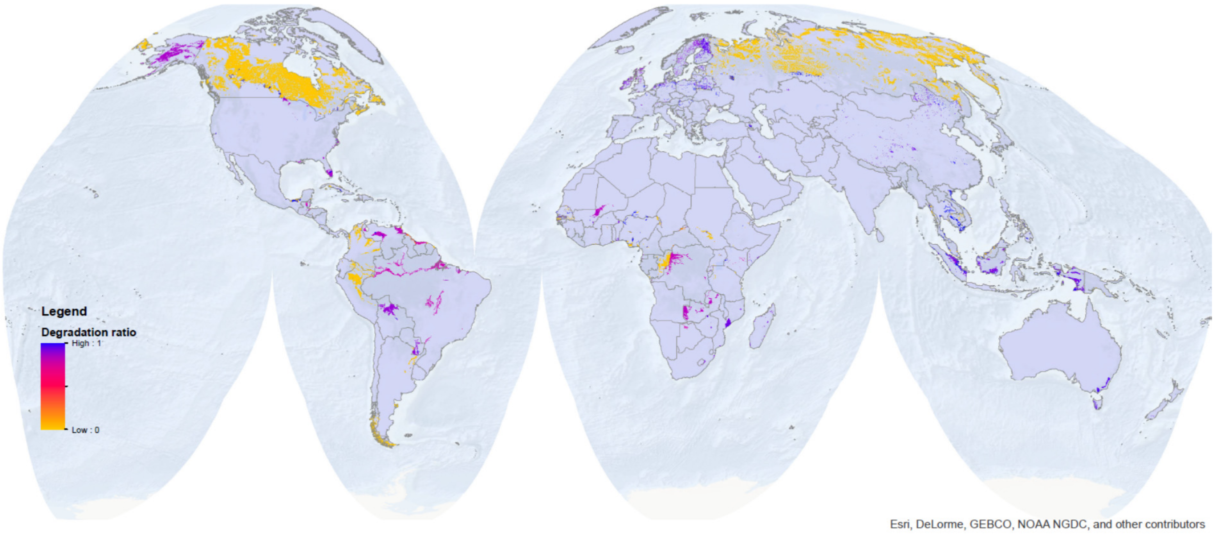

Supplementary Fig. 1. Degradation ratio of peatlands worldwide. Ratio 1 = fully degrading, ratio 0 = fully intact.

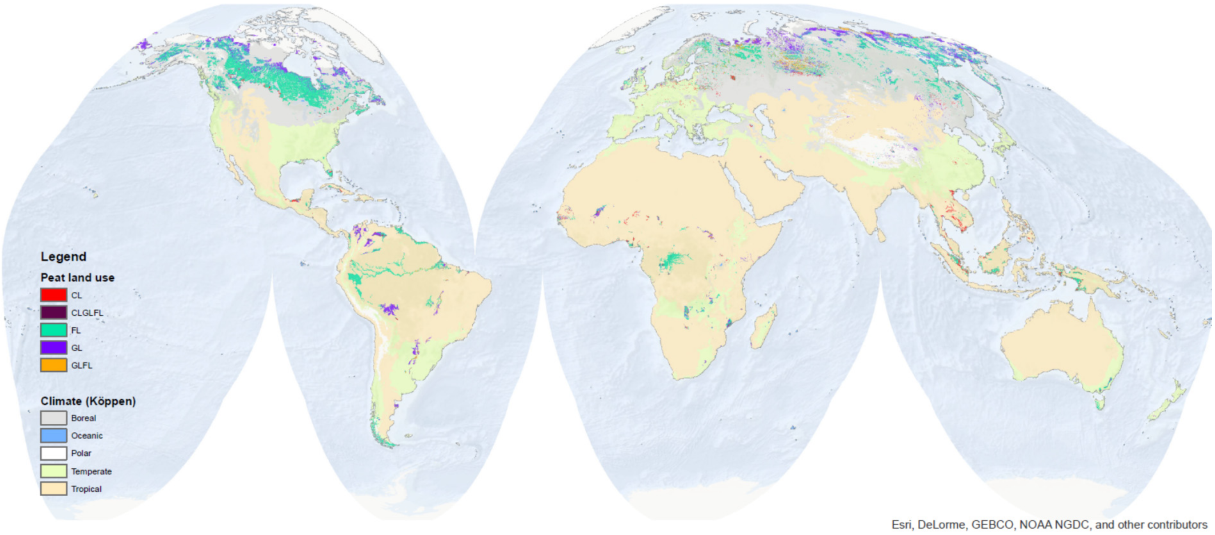

Supplementary Fig. 2. Compilation of land use types and climate zones used for assignment of emission factors of degrading peatlands per pixel.

Supplementary Table 1. Data mapping for reconciliation of the ESA-GLCM data with IPCC based data.

ESA-GCLM code	ESA-GCLM label	Assigned IPCC land-use class[1]
**11**	Post-flooding or irrigated croplands (or aquatic)	CL
**14**	Rainfed croplands	CL
**20**	Mosaic cropland (50–70%) / vegetation (grassland/shrubland/forest) (20–50%)	CL
**30**	Mosaic vegetation (grassland/shrubland/forest) (50–70%) / cropland (20–50%)	Average GL, FL and CL
**40**	Closed to open (>15%) broadleaved evergreen or semi-deciduous forest (>5 m)	FL
**50**	Closed (>40%) broadleaved deciduous forest (>5 m)	FL
**60**	Open (15–40%) broadleaved deciduous forest/woodland (>5 m)	FL
**70**	Closed (>40%) needleleaved evergreen forest (>5 m)	FL
**90**	Open (15–40%) needleleaved deciduous or evergreen forest (>5 m)	FL
**100**	Closed to open (>15%) mixed broadleaved and needleleaved forest (>5 m)	FL
**110**	Mosaic forest or shrubland (50–70%) / grassland (20–50%)	FL
**120**	Mosaic grassland (50–70%) / forest or shrubland (20–50%)	GL
**130**	Closed to open (>15%) (broadleaved or needleleaved, evergreen or deciduous) shrubland (<5 m)	FL
**140**	Closed to open (>15%) herbaceous vegetation (grassland, savannas or lichens/mosses)	GL
**150**	Sparse (<15%) vegetation	GL
**160**	Closed to open (>15%) broadleaved forest regularly flooded (semi-permanently or temporarily) – Fresh or brackish water	FL
**170**	Closed (>40%) broadleaved forest or shrubland permanently flooded – Saline or brackish water	FL
**180**	Closed to open (>15%) grassland or woody vegetation on regularly flooded or waterlogged soil – Fresh, brackish or saline water	Average FL and GL
**190**	Artificial surfaces and associated areas (urban areas >50%)	GL
**200**	Bare areas	not applicable
**210**	Water bodies	not applicable
**220**	Permanent snow and ice	not applicable
**230**	No data (burnt areas, clouds, etc.)	not applicable

[1] CL, cropland; FL, forest land; GL, grassland.

Supplementary Table 2. Emission factors (mean and range, aggregated from [1])

Climate	Land use	GHG emission factor ($t\ CO_2$-eq. $ha^{-1}\ a^{-1}$)
Boreal and Polar	CL	33.4 (26.5-40.6)
	CL+GL+FL	20.2 (-0.6-40.8)
	FL	2.9 (-0.6-7.1)
	GL	24.3 (12.2-36.5)
	GL+FL	13.6 (-0.6-36.7)
Temperate	CL	33.4 (27.4-42.9)
	CL+GL+FL	21.7 (7.5-43.6)
	FL	11 (8.3-17.1)
	GL	20.7 (7.8-33.8)
	GL+FL	15.8 (7.5-33.8)
Tropical	CL	52.1 (1.3-107.5)
	CL+GL+FL	51.8 (-0.6-107.5)
	FL	60.6 (-0.2-93.5)
	GL	42.7 (19.3-69.2)
	GL+FL	51.7 (-0.2-94.9)

Supplementary References

1. IPCC 2013 Supplement to the 2006 IPCC Guidelines for National Greenhouse Gas Inventories: Wetlands. (eds. Hiraishi, T. *et al.*) (2014).

2. Mu, Z., Huang, A., Ni, J. & Xie, D. Linking annual N_2O emission in organic soils to mineral nitrogen input as estimated by heterotrophic respiration and soil C/N ratio. *PLoS ONE* **9**, e96572 (2014).

3. Anshari, G.Z. *et al.* Drainage and land use impacts on changes in selected peat properties and peat degradation in West Kalimantan Province, Indonesia. *Biogeosciences* **7**, 3403-3419 (2010).

4. Yonebayashi, K., Pechayapisit, J., Vijarnsorn, P., Zahari, A.B. & Kyuma, K. Chemical alterations of tropical peat soils determined by Waksman's proximate analysis and properties of humic acids. *Soil Sci. Plant Nutr.* **40**, 435-444 (1994).

5. Gandaseca, S., Salimin, M.I. & Ahmed, O.H. Effect of cultivation in different age's oil palm plantation on selected chemical properties of peat swamp soils. *AFF* **3**, 6-9 (2014).

6. Melling, L., Tan, C.S.Y. Goh, K.J. & Hatano, R. Soil microbial and root respirations from three ecosystems in tropical peatland of Sarawak, Malaysia. *J. Oil Palm Res.* **25**, 44-57 (2013).

7. Könönen, M., Jauhiainen, J., Laiho, R., Kusin, K. & Vasander, H. Physical and chemical properties of tropical peat under stabilised land uses. *Mires and Peat* **16**, Article 08, 01–13 (2015).

8. Lawson, I.T., Jones, T.D., Kelly, T.J., Coronado, E.N.H. & Roucoux, K.H. The geochemistry of Amazonian peats. *Wetlands* **34**, 905-915 (2014).

9. Arai, H. *et al.* Land use change affects microbial biomass and fluxes of carbon dioxide and nitrous oxide in tropical peatlands. *Soil Sci. Plant Nutr.* **60**, 423-434 (2014).

10. Gandois, L., Cobb, A.R., Hei, I.C., Lim, L.B.L., Salim, K.A. & Harvey, C.F. Impact of deforestation on solid and dissolved organic matter characteristics of tropical peat forests: implications for carbon release. *Biogeochemistry* **114**, 183-199 (2013).

11. Sjögersten, S., Cheesman, A.W., Lopez, O. & Turner, B.L. Biogeochemical processes along a nutrient gradient in a tropical ombrotrophic peatland. *Biogeochemistry* **104**, 147-163 (2011).

12. Chimner, R.A. & Ewel, K.C. A tropical freshwater wetland: II. Production, decomposition, and peat formation. *Wetl. Ecol. Manag.* **13**, 671-684 (2005).

13. Hadi, A., Inubushi, K., Furukawa, Y., Purnomo, E., Rasmadi, M. & Tsuruta, H. Greenhouse gas emissions from tropical peatlands of Kalimantan,Indonesia. *Nutr. Cycl. Agroecosys.* **71**, 73-80 (2005).

14. Adame, M.F., Santini, N.S., Tovilla, C., Vázquez-Lule, A., Castro, L. & Guevara, M. Carbon stocks and soil sequestration rates of tropical riverine wetlands. *Biogeosciences* **12**, 3805-3818 (2015).

15. Batjes, N.H. Harmonized soil profile data for applications at global and continental scales: updates to the WISE database. *Soil Use Manag.* **25**, 124-127 (2009).

16. Pajunen, H. Physical and chemical properties of peat in Rwanda, Central Africa. *Geological Survey of Finland Bulletin* **394**, 1-61 (1997).

17. Hope, G.S. Peat in the mountains of New Guinea. *Mires and Peat* **15**, 13-13 (2014).

18. Hribljan, J.A*, et al.* Carbon storage and long-term rate of accumulation in high-altitude Andean peatlands of Bolivia. *Mires and Peat* **15**, 12-12 (2014).
