# Supplementary material for: The underappreciated potential of peatlands in global climate change mitigation strategies
Source: Nat Commun. 2018 Mar 14;9:1071. doi: 10.1038/s41467-018-03406-6 (PMC5851997; doi:10.1038/s41467-018-03406-6)
Supplement: Supplementary file 3 — Description of Additional Supplementary Files [file 41467_2018_3406_MOESM3_ESM.pdf]

Description of Additional Supplementary Files

File Name: Supplementary Data 1
Description: Organic carbon, nitrogen, and soil C/N ratios of tropical peatlands.
