# Supplementary material for: The underappreciated potential of peatlands in global climate change mitigation strategies
Source: Nat Commun. 2018 Mar 14;9:1071. doi: 10.1038/s41467-018-03406-6 (PMC5851997; doi:10.1038/s41467-018-03406-6)
Supplement: Supplementary file 4 — Supplementary Data 1 [file 41467_2018_3406_MOESM4_ESM.pdf]

Organic carbon, nitrogen, and soil C/N ratios of tropical peatlands.

Source	Wise ID	Country	Land use	pH	Soil organic C (%)	Soil N (%)	C/N	Comment
Mu, Huang [2]		Malaysia	forest	3.4	44.7	2	23.4	
Mu, Huang [2]		Malaysia	forest	3.6	47.8	1.8	27.2	
Mu, Huang [2]		Malaysia	forest	3.6	44.6	2	22.6	
Mu, Huang [2]		Indonesia	forest	3.4	57.9	0.9	63.6	
Mu, Huang [2]		Indonesia	forest	3	54.3	1.7	31.9	
Mu, Huang [2]		Indonesia	forest	3.4	58.2	0.8	69.3	
Mu, Huang [2]		Indonesia	cropland	3.8	48.6	1	50.6	
Mu, Huang [2]		Indonesia	cropland	3.8	61.3	1.5	40.9	
Mu, Huang [2]		Indonesia	forest	3.2	48.6	1.6	30.6	
Anshari, Afifudin [3]		Indonesia	forest	3.6	51.8	1.8	28	
Anshari, Afifudin [3]		Indonesia	forest	3.6	52.1	2.3	22.3	
Anshari, Afifudin [3]		Malaysia	reclaimed coastal swamp	no data	56.3	2.1	27.3	
Anshari, Afifudin [3]		Malaysia	reclaimed coastal swamp	no data	56.3	1.4	41.2	
Yonebayashi, Pechayapisit [4]		Thailand	reclaimed coastal swamp	no data	57.3	2.4	24	
Gandaseca, Salimin [5]		Malaysia	oil palm	3.7	55.6	0.8	67.8	
Gandaseca, Salimin [5]		Malaysia	oil palm	3.2	55.7	1.7	33.5	
Gandaseca, Salimin [5]		Malaysia	oil palm	4.1	54.7	0.9	59.9	
Melling, Tan [6]		Malaysia	forest	3.6	48.2	1.6	30.8	0–50 cm
Melling, Tan [6]		Malaysia	oil palm	3.3	45.4	1.6	27.8	0–50 cm
Melling, Tan [6]		Malaysia	sago	3.6	45.6	1.9	24.7	0–50 cm
Melling, Tan [6]		Malaysia	forest	1.9	57.5	1.9	29.7	
Melling, Tan [6]		Malaysia	forest	1.9	56.4	1.9	29.7	
Melling, Tan [6]		Malaysia	forest	1.8	55.5	1.8	30.3	
Könönen, Jauhiainen [7]		Indonesia	forest	3.4	55.5	1.6	34.7	
Könönen, Jauhiainen [7]		Indonesia	forest	3.7	57.3	1.6	36	
Könönen, Jauhiainen [7]		Indonesia	forest	3	61.5	0.9	71.5	
Könönen, Jauhiainen [7]		Indonesia	forest	3.1	55.5	1.9	29.5	
Könönen, Jauhiainen [7]		Indonesia	forest	2.7	63.8	0.9	71.6	
Könönen, Jauhiainen [7]		Indonesia	forest	2.9	59.3	0.9	68.9	
Könönen, Jauhiainen [7]		Indonesia	forest	3.1	60.4	0.7	82.7	
Könönen, Jauhiainen [7]		Indonesia	degraded	3.6	58.7	0.9	69.1	
Könönen, Jauhiainen [7]		Indonesia	degraded	3.3	58.5	0.8	73.2	
Könönen, Jauhiainen [7]		Indonesia	degraded	3	58.8	0.8	78.4	
Könönen, Jauhiainen [7]		Indonesia	degraded	3.4	57.9	0.7	82.7	
Könönen, Jauhiainen [7]		Indonesia	cropland	3.3	58.6	0.9	66.6	
Könönen, Jauhiainen [7]		Indonesia	cropland	3.4	58.3	0.8	75.7	
Könönen, Jauhiainen [7]		Indonesia	cropland	3.4	59.6	0.9	67.7	
Könönen, Jauhiainen [7]		Indonesia	cropland	3.5	58.2	0.7	85.6	
Lawson, Jones [8]		Peru	natural swamp	no data	46.5	1.6	28.7	upper peat
Arai, Hadi [9]		Indonesia	forest	3.6	50.4	1.3	37.9	
Arai, Hadi [9]		Indonesia	forest	3.8	49.4	1.8	27.1	
Arai, Hadi [9]		Indonesia	forest	3.8	53.7	1.6	34.4	
Arai, Hadi [9]		Indonesia	forest	3.8	56.8	1.3	42.7	
Arai, Hadi [9]		Indonesia	cropland	4.2	32.5	1.1	30.7	
Arai, Hadi [9]		Indonesia	cropland	3.7	43.7	1.3	33.6	
Arai, Hadi [9]		Indonesia	cropland	4.3	37.2	1	37.2	
Arai, Hadi [9]		Indonesia	cropland	5.1	33.6	1.1	30	
Gandois, Cobb [10]		Brunei	forest	3.9	52.3	2.1	24.9	
Gandois, Cobb [10]		Brunei	forest	4	50.6	1.9	26.6	

Gandois, Cobb [10]		Brunei	forest	4.1	55	1.8	30.6
Gandois, Cobb [10]		Brunei	forest	4.1	51.5	1.9	27.1
Gandois, Cobb [10]		Brunei	forest	4.1	54.7	1.8	30.4
Gandois, Cobb [10]		Brunei	deforested	4.8	50.2	1.6	31.4
Gandois, Cobb [10]		Brunei	deforested	3.9	46.7	1.9	24.6
Gandois, Cobb [10]		Brunei	deforested	4.1	50.2	2.3	21.8
Gandois, Cobb [10]		Brunei	deforested	4.2	51.1	2.4	21.3
Gandois, Cobb [10]		Brunei	deforested	4.4	47.4	2.8	16.9
Gandois, Cobb [10]		Brunei	deforested	3.7	51.1	2.9	17.6
Gandois, Cobb [10]		Brunei	deforested	3.6	52	2.6	20
Sjögersten, Cheesman [11]		Panama	natural swamp	3.8	49	2.4	20.6
Sjögersten, Cheesman [11]		Panama	natural swamp	3.7	48.1	2.6	18.6
Sjögersten, Cheesman [11]		Panama	natural swamp	3.9	48.9	2.7	18.4
Sjögersten, Cheesman [11]		Panama	natural swamp	4	49.7	2.6	18.8
Sjögersten, Cheesman [11]		Panama	natural swamp	4	50.2	2.5	20.1
Chimner and Ewel [12]		Micronesia	forest	6.4	19.9	1.2	16.6
Chimner and Ewel [12]		Micronesia	forest	5.9	47	2.3	20.1
Hadi, Inubushi [13]		Indonesia	forest	3.3	13.1	0.6	22.6
Hadi, Inubushi [13]		Indonesia	paddy field	3.4	20.2	0.6	31.5
Hadi, Inubushi [13]		Indonesia	cropland	3.3	14.3	0.5	28.7
Hadi, Inubushi [13]		Indonesia	forest	3.2	31.7	0.7	46.6
Hadi, Inubushi [13]		Indonesia	paddy field	3.7	37.7	0.7	55.4
Hadi, Inubushi [13]		Indonesia	cropland	3.5	61.6	1.2	50.5
Hadi, Inubushi [13]		Indonesia	forest	4.5	18	0.7	24.4
Hadi, Inubushi [13]		Indonesia	forest	4.4	46.5	1.8	26.1
Hadi, Inubushi [13]		Indonesia	paddy field	4.4	50.7	1.5	33.2
Hadi, Inubushi [13]		Indonesia	cropland	4.6	57.4	1.3	43.5
Adame, et al. [14]		Mexico	peat swamp forest	no data	21.8	1.3	16.8
Batjes [15]	BD0017	Bangladesh	unknown	5.5	40.7	2.1	19.5
Batjes [15]	CD0130	Democratic Republic Of The Congo	unknown	6.2	19.3	0.3	66
Batjes [15]	CO0041	Colombia	unknown	4.5	20.6	1.4	14.6
Batjes [15]	CO0057	Colombia	unknown	4.7	33.3	1.6	20.3
Batjes [15]	CO0058	Colombia	unknown	4.8	42.6	1.6	27
Batjes [15]	CO0060	Colombia	unknown	4.4	45.4	1.3	35.3
Batjes [15]	CO0062	Colombia	unknown	4.4	32.1	1.9	17
Batjes [15]	GY0024	Guyana	unknown	3.7	16.2	0.7	24.6
Batjes [15]	GY0025	Guyana	unknown	3.7	29.5	0.6	48.7
Pajunen [16]		Rwanda	natural swamp	5	52.2	1.9	27.5
Pajunen [16]		Rwanda	natural swamp	5	42.6	1.9	22.4
Pajunen [16]		Rwanda	natural swamp	no data	52	1.6	32.5
Pajunen [16]		Rwanda	natural swamp	no data	53.9	1.8	29.9
Pajunen [16]		Rwanda	natural swamp	no data	50.2	1.7	29.5
Pajunen [16]		Rwanda	cropland	4.9	57.8	1.8	32.1
Pajunen [16]		Rwanda	cropland	4.9	54.9	1.9	28.9
Pajunen [16]		Rwanda	cropland	4.9	54.1	1.8	30.1
Pajunen [16]		Rwanda	cropland	4.9	56.5	1.4	40.4
Pajunen [16]		Rwanda	natural bog	3.4	55.9	1.3	43
Pajunen [16]		Rwanda	abandoned peat extraction site	4.7	52.6	1.6	32.9
Pajunen [16]		Rwanda	peat extraction site	5.5	54.5	1.6	34.1
Hope [17]		PNG[1]	natural peatland	4	13.4	0.9	14.9
Hope [17]		PNG[1]	natural peatland	5.8	21	0.5	42
Hope [17]		PNG[1]	natural peatland	6.7	35.4	1.4	25.3
Hope [17]		PNG[1]	natural peatland	6.4	31.2	1.1	28.4
Hope [17]		PNG[1]	natural peatland	5.4	16.8	0.9	18.7

Hope [17]	PNG[1]	natural peatland	5.7	18.8	1.3	14.5
Hope [17]	PNG[1]	natural peatland	6	35.2	0.9	39.1
Hope [17]	PNG[1]	natural peatland	3.4	22.7	2.3	10
Hope [17]	PNG[1]	natural peatland	6.1	37	1.6	23.1
Hope [17]	PNG[1]	natural peatland	5.9	29.5	1	29.5
Hope [17]	PNG[1]	natural peatland	5.6	13.9	0.8	17.4
Hope [17]	PNG[1]	natural peatland	5.6	15.8	0.8	19.8
Hribljan, Cooper [18]	Bolivia	grazed	no data	23.9	1.1	21.7
Hribljan, Cooper [18]	Bolivia	grazed	no data	47.8	2	23.9
n			**102**	**112**	**112**	**112**
**mean**			**4.1**	**45.3**	**1.5**	**35**
**median**			**3.8**	**50.2**	**1.6**	**29.7**
**upper 95% CI**			**4.3**	**47.9**	**1.6**	**38.4**
**lower 95% CI**			**3.9**	**42.7**	**1.4**	**31.7**
**1 SD**			**1**	**13.9**	**0.6**	**17.8**

[1] Papua New Guinea
